# Upregulation of UDP-Glucuronosyltransferases 1a1 and 1a7 Are Involved in Altered Puerarin Pharmacokinetics in Type II Diabetic Rats

**DOI:** 10.3390/molecules23061487

**Published:** 2018-06-20

**Authors:** Songtao Dong, Maofan Zhang, Huimin Niu, Kunyu Jiang, Jialei Jiang, Yinglin Ma, Xin Wang, Shengnan Meng

**Affiliations:** Department of Pharmaceutics, School of Pharmacy, China Medical University, Shenyang 110122, China; dongsongtao8886@163.com (S.D.); mfzhang@cmu.edu.cn (M.Z.); 15804066249@163.com (H.N.); jiangkunyu87@163.com (K.J.); 15804002893@163.com (J.J.); ylmacmu@163.com (Y.M.); WangXinCMU@126.com (X.W.)

**Keywords:** puerarin, pharmacokinetics, diabetes, *Ugt1a1*, *Ugt1a7*

## Abstract

Puerarin is an isoflavonoid extracted from *Pueraria lobata* roots, and displays a broad range of pharmacological activities, including antidiabetic activity. However, information about the pharmacokinetics of puerarin in diabetics is scarce. This study was conducted to investigate the difference in pharmacokinetic effects of puerarin in normal rats and rats with diabetes mellitus (DM), and the mechanism involved. DM was induced by a combined high-fat diet (HFD) and streptozotocin (STZ) injection. Plasma concentrations of puerarin in DM, HFD, and control rats were determined after intravenous (20 mg/kg) and oral administration (500 mg/kg) of puerarin, and pharmacokinetic parameters were estimated. The messenger RNA (mRNA) and protein expression levels of *Ugt1a1* and *Ugt1a7* in rat livers and intestines were measured using qRT-PCR and western blot, respectively. The area under the concentration–time curve and the clearance of puerarin in the DM rats statistically differed from those in the control rats (*p* <0.05) with both administration routes. The hepatic and intestinal gene and protein expressions of *Ugt1a1* and *Ugt1a7* were significantly increased in the DM rats (*p* <0.05). Therefore, the metabolic changes in diabetes could alter the pharmacokinetics of puerarin. This change could be caused by upregulated uridine diphosphate (UDP)-glucuronosyltransferase activity, which may enhance puerarin clearance, and alter its therapeutic effects.

## 1. Introduction

**Figure 1:**
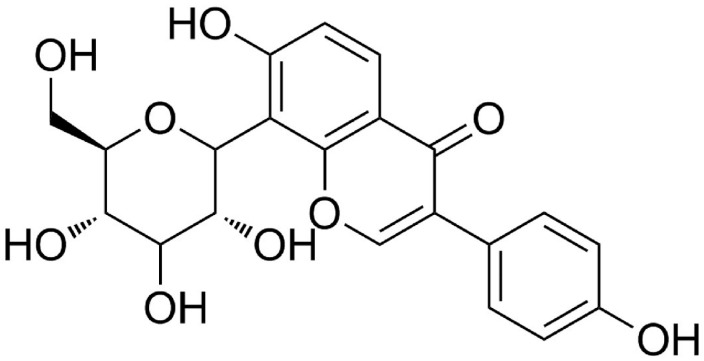
Chemical structure of puerarin.

Puerarin (7,4′-dihydroxyisoflavone-8-*β*-glucopyranoside) (Figure 1) is the major bioactive isoflavone isolated from the roots of the wild leguminous creeper, *Pueraria lobata* (kudzuvine root, also known as Gegen) [1]. In China and other Asian countries, it is commonly used for the prevention and treatment of cardiovascular and cerebrovascular diseases [2], including hypertension, heart failure, arteriosclerosis, and myocardial ischemia. Puerarin was reported to exert protective effects against inflammation [3], hyperlipidemia [4], and oxidative stress [5]; it was even reported to improve vascular insulin resistance [6]. Considering its strong pharmacologic effects, beneficial functions, and wide availability, puerarin is widely used in clinical applications.

Diabetes mellitus (DM) is one of the most common chronic diseases that create great impacts on human health. DM patients may require the use of different kinds of drugs to control their blood glucose levels and diabetic complications. Cardiovascular diseases are often the leading causes of death and disability in patients with Type II DM [7,8], and puerarin is often used as one of the conventional agents to treat such complications of DM. Moreover, both clinical trials and animal studies demonstrated that puerarin can improve diabetic symptoms and complications by reducing blood glucose levels [9], enhancing glucose uptake [10], protecting the pancreatic beta cells [11], and lowering insulin resistance [12]. Puerarin has the potential to play an important role in the treatment of DM.

It is very important for us to define the pharmacokinetic procession of medicines in disease states in order to apply them much more safely, reasonably, and effectively. Previous pharmacokinetic studies of puerarin were implemented in rat models of asthma [13], nonalcoholic fatty liver disease (NAFLD) [14], and endometriosis [15]. These pharmacokinetic data could help guide the medicinal application of puerarin in each disease. Puerarin is now very active in therapeutic regimens for DM, but there is no research to lend insight into the pharmacokinetics of puerarin conducted in the state of DM. Furthermore, the pathogenesis and pathophysiology of DM may alter the expressions and functions of drug-metabolizing enzymes, impacting drug pharmacokinetic behavior, efficacy, and toxicity. Published literature shows that the metabolism of puerarin in vivo involves both phase I and phase II catalytic reactions [16,17]. Based on the amount and type of metabolites, the phase II biotransformation of puerarin can be more important than that of phase I. Glucuronidation of puerarin is the main phase II reaction, predominantly involving *UGT1A1* and *UGT1A9* in human beings [18]. Because *UGT1A9* is a pseudogene, there is no expression in rats, and its function in rats is considered to be replaced by *Ugt1a7* [19].

In the presented study, we investigated the pharmacokinetics of puerarin, as well as the changes in messenger RNA (mRNA) and protein expression of *Ugt1a1* and *Ugt1a7* in the liver and intestines of a type II DM rat model. Our findings provide important new insights into the pharmacokinetic characteristics of puerarin in DM, which are critical to guide the use of puerarin in treating patients with DM.

## 2. Results

### 2.1. Validation of Type II Diabetic Rat Model

Parameters such as triglyceride, total cholesterol, blood glucose, homeostasis model assessment of insulin resistance (HOMA-IR), and body weight were measured in the control (CON), high-fat diet (HFD), and DM rats (Table 1). The blood levels of glucose, triglycerides, and total cholesterol in DM rats were markedly higher than those in the CON and HFD rats. The DM rats also had significantly increased HOMA-IR values, accompanied by reduced body weights (Table 1) and development of diabetic symptoms, such as polyuria, polyphagia, and polydipsia. These indexes were similar to those of type II diabetic patients, indicating that the DM rats may be considered type II diabetic rats [20]. The HFD rats showed some diabetic symptoms and higher body weights than the CON rats did, but their glucose levels were similar to those of the CON rats. These indexes of the HFD rats were similar to those of the pre-diabetic state in humans.

### 2.2. Assay Validation

**Figure 2:**
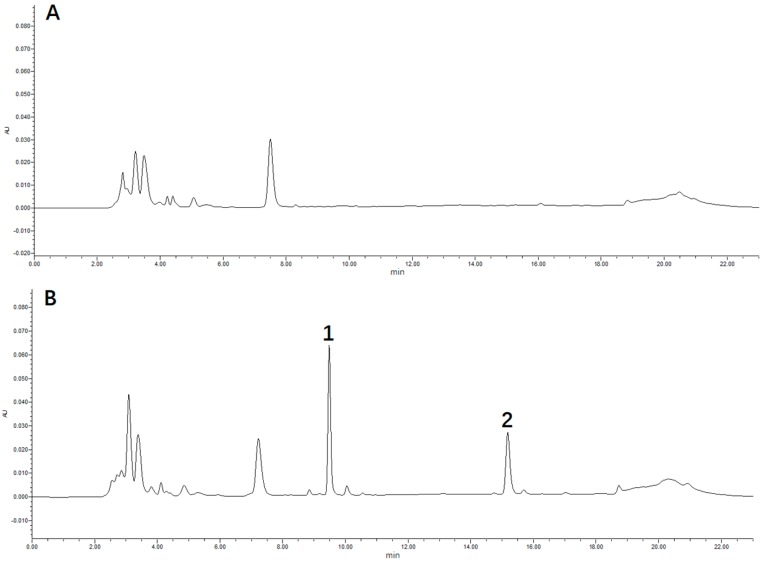

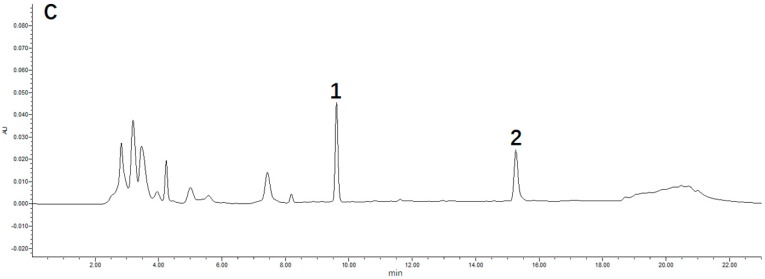
Typical chromatograms: (**A**) blank rat plasma; (**B**) blank rat plasma spiked with puerarin and methyl *p*-hydroxybenzoate (internal standard, IS); (**C**) rat plasma sample. Peak 1: puerarin; Peak 2: IS.

A rapid and sensitive method was developed for the determination of puerarin concentration in rat plasma using HPLC. The typical retention times of puerarin and methyl *p*-hydroxybenzoate (internal standard, IS) were 9.0 min and 15.5 min, respectively, and the chromatographic peaks were sharp and symmetrical (Figure 2). Calibration curves were constructed daily with adequate linearity over the concentration range of 0.1–100 μmol/L for puerarin, and they could be modeled by the regression equation: *Y* = 0.0849*X* + 0.1011 (R^2^ = 0.9997). The lower limit of quantification (LLOQ) was determined as 0.1 μmol/L by analyzing five replicates of the lowest standard on the calibration curve. The intra-day and inter-day accuracy and precision were determined by replicate analyses of the quality control (QC) samples (*n* = 5) at three concentrations (low, medium, and high), and ranged within the acceptance limit of 15% (Table 2). The extraction recoveries of puerarin in rat plasma from three different QC samples (0.2, 5, and 80 μmol/L; *n* = 5) were 90.81%, 91.62%, and 93.61%, respectively. The matrix effects for puerarin and IS ranged from 85% to 115%, indicting no significant matrix effect that interfered with puerarin determination in the rat blood. The analytes were stable in rat plasma at room temperature, 4 °C, and 25° C for 24 h, and −40 °C for 30 days (Table 3). The puerarin in rat plasma was also stable after three freeze–thaw cycles at −40 °C (Table 3).

### 2.3. Pharmacokinetic Properties of Puerarin

**Figure 3:**
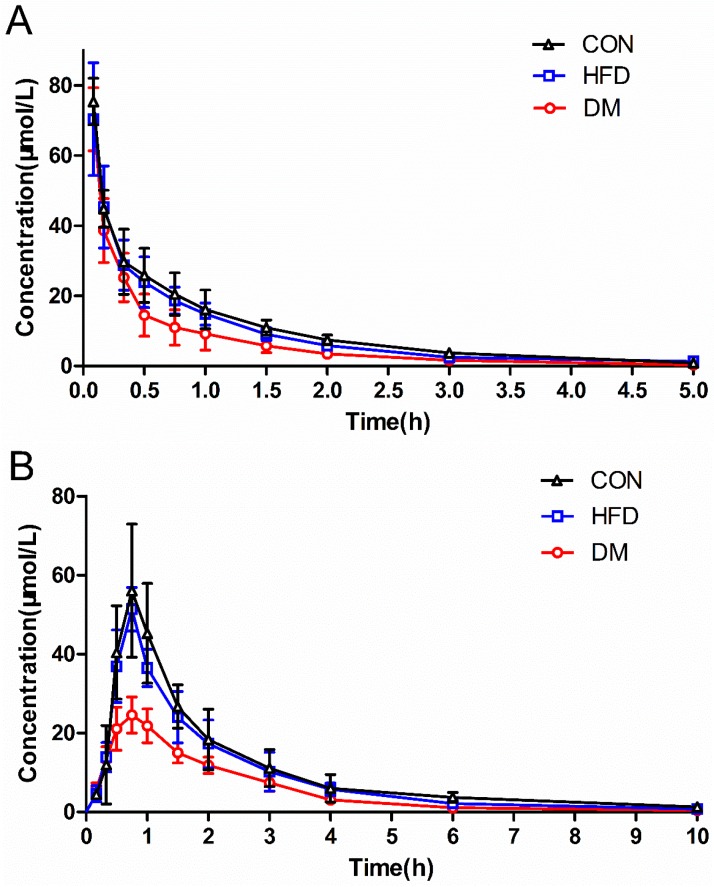
Plasma concentration–time curves for puerarin in rat plasma after intravenous (at 20 mg/kg, **A**) and oral administration (at 500 mg/kg, **B**) to diabetes mellitus (DM), high-fat diet (HFD), and control (CON) rats. Each point represents the mean ± SD (*n* = 7).

The puerarin concentrations in the plasma of DM, HFD, and CON rats, and the plasma concentration–time profiles after different routes of administration are shown in Figure 3. The mean concentrations of puerarin in the DM rats were significantly lower than those in the CON rats whether administered intravenously (i.v.) or orally (p.o.). The mean concentrations of puerarin in the HFD and CON groups were not significantly different whether administered intravenously or orally. The corresponding pharmacokinetic parameters are summarized in Table 4 and Table 5. The pharmacokinetic parameters, area under the curve (AUC), mean residence time (MRT), and clearance (CL_z_) from the CON and DM rats following intravenous dosing were significantly different (*p* <0.05 or *p* <0.01). For the oral route, the AUC, C_max_, and half time (t_1/2z_) of the analytes were significantly lowered in the DM rats (*p* <0.01), whereas the clearance divided by the absorption fraction (CL_z_/F) and the apparent volume of distribution divided by the absorption fraction (V_z_/F) values were significantly higher in the CON rats (*p* < 0.05). However, the differences between the DM and CON rats were greater with oral dosing than with intravenous dosing. These results indicate that the systemic exposure of the analytes after oral administration of puerarin was remarkably decreased in the DM rats.

### 2.4. Ugt1a1 and Ugt1a7 mRNA Levels in the Rat Liver and Intestine

**Figure 4:**
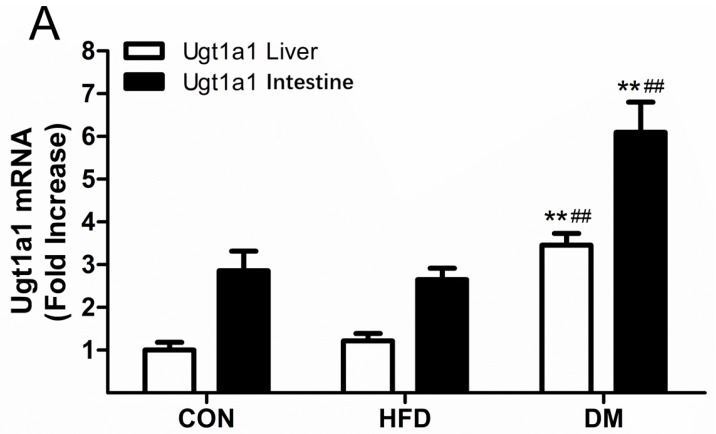

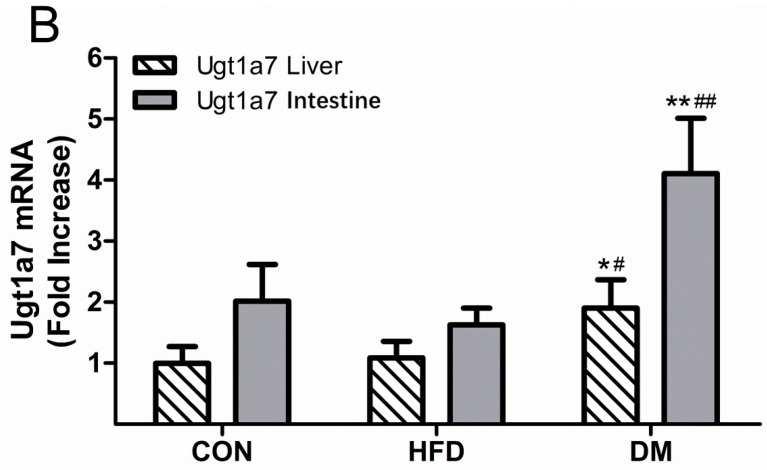
Messenger RNA (mRNA) expression levels of *Ugt1a1* (**A**) and *Ugt1a7* (**B**) in the liver and intestines of DM, HFD, and CON rats. Data represent the mean ± SD, *n* = 7 rats, * *p* <0.05, ** *p* <0.01 in DM versus CON rats; ^#^ *p* <0.05, ^##^ *p* <0.01 in DM versus HFD rats.

The metabolism of puerarin is mainly catalyzed by phase II metabolic enzymes, where uridine diphosphate (UDP)-glucuronosyltransferase (UGT) 1A1 (*Ugt1a1*) and Ugt1a7 are the dominate isoforms in rats. In order to explore their possible impacts on the pharmacokinetics of puerarin in CON, HFD, and DM rats, we quantified the gene expressions of *Ugt1a1* and *Ugt1a7* in the liver and intestines of rats. Figure 4 illustrates the relative mRNA levels of *Ugt1a1* and *Ugt1a7* of the rats. The mRNA expressions of *Ugt1a1* in the liver and intestines of DM rats were approximately 3.38-fold and 2.81-fold higher than those of CON rats, and were approximately 1.34-fold and 1.29-fold higher than those of HFD rats, respectively (Figure 4A). The *Ugt1a7* levels showed similar trends, with relative mRNA levels in the DM rats approximately 1.90-fold and 1.75-fold higher than those in the CON rats, and approximately 2.04-fold and 2.52-fold higher than those in HFD rats in the liver and intestines, respectively (Figure 4B). These results indicate that diabetes can upregulate the mRNA expressions of *Ugt1a1* and *Ugt1a7* in rats.

### 2.5. Ugt1a1 and Ugt1a7 Protein Expression in the Rat Liver and Intestine

**Figure 5:**
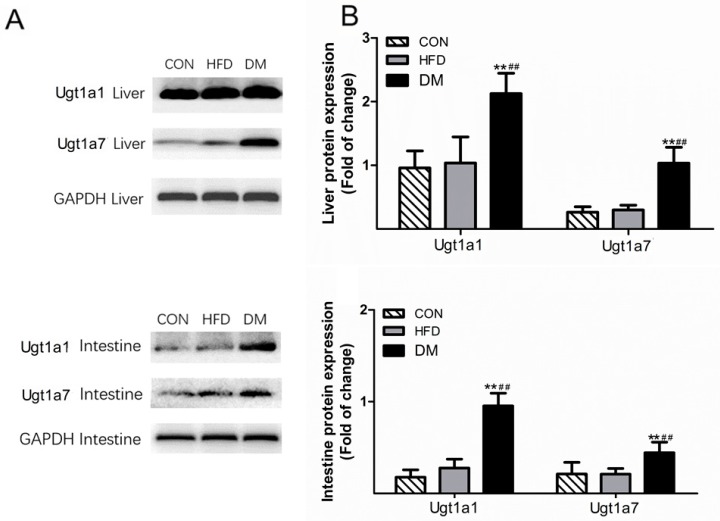
Expression of hepatic and intestinal uridine diphosphate (UDP)-glucuronosyltransferase (UGT) 1A1 (Ugt1a1) and Ugt1a7 proteins in DM, HFD, and CON rats. Data represent the mean ± SD, *n* = 3 rats, * *p* <0.05, ** *p* <0.01 in DM versus CON rats; ^#^ *p* <0.05, ^##^ *p* <0.01 in DM versus HFD rats.

According to the results of western blots, the hepatic and intestinal expressions of the Ugt1a1 and Ugt1a7 proteins were markedly increased in DM rats when compared with those in HFD and CON rats (Figure 5). The protein expression levels of Ugt1A1 in the liver and intestines of DM rats were approximately 2.02-fold and 5.43-fold higher than those of the CON rats, and 2.05-fold and 3.45-fold higher than those of HFD rats, respectively. Similar trends were found with Ugt1a7, where its protein expression in DM rats was approximately 3.96-fold and 2.07-fold higher than that in CON rats, and 3.49-fold and 2.09-fold higher than that in HFD rats in the liver and intestines, respectively. These results are consistent with the mRNA expression levels, demonstrating that the protein expressions of Ugt1a1 and Ugt1a7 in rat liver and intestine were enhanced under the diabetic pathology.

## 3. Discussion

Puerarin was approved by the China Food and Drug Administration (CFDA) as an adjuvant treatment for cardiovascular and cerebrovascular diseases; it is often used in China by diabetic patients to treat their microvascular and macrovascular complications. Many pathological conditions, such as DM, could potentially affect the pharmacokinetics of drugs [20,21]. This study was first undertaken to characterize the altered pharmacokinetics of puerarin after its oral and intravenous administration in type II diabetic rats. Diabetes is categorized as either type I (insulin dependent) or type II (non-insulin dependent). Because type II is the most common form of diabetes, comprising ~90% of DM cases, more research should be focused on type II diabetes. The type II diabetic model used in this study was induced through combined HFD and low-dose streptozotocin (STZ). The rats were hyperglycemic, with increased HOMA-IR, which mimicked the physiological characteristics of human type II diabetes. In addition to a normal control, an HFD control was also used in this study, since an HFD can induce metabolic syndromes, such as hyperlipidemia and insulin resistance [22].

In our study, we used 20 mg/kg and 500 mg/kg of puerarin for i.v. and p.o. administration to rats, respectively. These doses we set for rats were based on the clinical daily doses of puerarin, as well as published articles on the biological characteristics of puerarin [23,24,25]. In addition, we calculated doses in our previous studies on puerarin pharmacokinetics in normal and hepatofibrosis rats (manuscript in press), and the results indicated that the doses we adopted in our current study were reasonable.

For the pharmacokinetic parameters of puerarin, including AUC_0–t_, AUC_0–∞_, CL_z_ or CL_z_/F, and V_z_/F, there were significant differences between the CON and DM rats (*p* <0.05 or *p* <0.01). The AUC values were decreased, and the CL and V values were increased, which indicates that the pathological state might reduce the absorption of puerarin and/or enhance its metabolism. Especially with the oral route, the parameters C_max_ and t_1/2z_ were remarkably decreased in the DM rats (*p* <0.05), substantially lowering the systemic exposure (AUC and C_max_) to puerarin. Therefore, care should be taken with regard to the efficacy of puerarin when its products are used in clinical treatments. Additional studies are needed to gain a better understanding of the molecular mechanism underlying decreased puerarin absorption after oral administration.

We performed a comparative study of the pharmacokinetics of puerarin between normal and DM rats. The CL and AUC of both groups displayed remarkable differences. Since the AUC is equal to the dose of administration divided by the CL (for intravenous) or the CL_z_/F (for oral), the changes in CL would have an influence on the values of AUC. The expression of drug-metabolizing enzymes and their activities greatly affect the CL, so we decided to analyze this further. It was determined that *UGT1A1* and *UGT1A9* are the dominant isoforms of puerarin in humans; however, human and animal species have different UGT gene systems [16]. *UGT1A9* is functional in humans, whereas rat *Ugt1a9* is a pseudogene, and *Ugt1a7* compensated for the functions of *Ugt1a9* in rats [17,25]. For this reason, we investigated the mRNA and protein expressions of *Ugt1a1* and *Ugt1a7* in rat liver and intestines to predict the influence of the expression of drug-metabolizing enzymes on the pharmacokinetics of puerarin. The results indicated that the pathological state of diabetes could significantly increase the mRNA and protein levels of *Ugt1a1* and *Ugt1a7* in rats. These alterations were consistent with the research of Xie et al. [26] on diabetic rats. The enhanced enzymatic activities of Ugt1a1 and Ugt1a7 may have substantial effects on the pharmacokinetics of puerarin (such as CL and AUC), and ultimately, on its clinical efficacy. In further studies, we will focus on transcriptional regulation in diabetic rats.

In conclusion, to the best of our knowledge, our study demonstrated for the first time that the pharmacokinetic behaviors of puerarin were significantly changed in type II DM rats. This might be due to the enhanced puerarin metabolism achieved via upregulated *Ugt1a1* and *Ugt1a7* mRNA and protein levels in rat liver and intestines, which could play an important role in the pharmacokinetic alterations in puerarin. This study provides valuable information for the use of puerarin in the clinical treatment of diabetic patients.

## 4. Materials and Methods

### 4.1. Chemicals

Puerarin (99.3% purity) was purchased from Chengdu Longquan High-Tech Natural Pharmaceutical Co., Ltd. (Chengdu, China). Methyl *p*-hydroxybenzoate (IS) was obtained from Meloneparma Biotechnology Co., Ltd. (Dalian, China). Heparin sodium and streptozotocin (STZ) were purchased from Sigma Chemical Co., Ltd. (St Louis, MO, USA). The glucose test kit was purchased from Roche Co., Ltd. (Shanghai, China). Triglyceride and total cholesterol kits were from Beijing BHKT Clinical Reagent Co., Ltd Beijing, China. Rat insulin ELISA assay kits were supplied by Mercodia Co., Ltd. (Uppsala, Sweden). The GoScript reverse transcriptase kit and GoTaq^®^ qPCR Master Mix kit were purchased from Promega Co., Ltd. (Beijing, China). TRIzol was purchased from Invitrogen Co., Ltd. (Shanghai, China). UGT primers were synthesized by Beijing Dingguo Changsheng Biotechnology Co., Ltd. (Beijing, China). Anti-*Ugt1a1* (abs-134323) and Anti-*Ugt1a7* (sc-377075) were purchased from Absin Bioscience Inc. (Shanghai, China) and Santa Cruz Co., Ltd. (Shanghai, China), respectively. Methanol and acetonitrile (HPLC grade) were purchased from Concord Technology Co., Ltd. (Tianjin, China). All other reagents were analytical grade and were commercially available.

### 4.2. Animals

Male Sprague/Dawley rats, weighing 180–220 g, were obtained from the Laboratory Animal Center of the China Medical University (Shenyang, China). The rats were maintained under environmentally controlled conditions, at a temperature 22 ± 2 °C, a relative humidity of 50 ± 5%, and a 12-h light/dark cycle. Rats were allowed free access to food and water for at least one week prior to the initiation of experiments. The rats were fasted overnight prior to dosing, but had free access to water. The use of animals in the presented study was permitted by the Ethics Committee of the China Medical University, and all animal studies were carried out according to the National Institutes of Health (NIH) Guide for the Care and Use of Laboratory Animals (8th edition, revised 2011).

### 4.3. STZ-Induced Type II Diabetic Rats

The type II diabetic model was developed using a combination of a HFD and a low-dose STZ injection [18]. Briefly, rats were randomized into three groups: the CON group, the HFD group, and the DM group. The CON rats received a standard diet, whereas both HFD and DM rats were fed high-fat food, which consisted of 63.5–66.5% normal chow, 1% cholesterol, 0.5% sodium cholate, 10% yolk, 12–15% lard, and 10% sucrose. DM rats were given an intraperitoneal injection of STZ (35 mg/kg, dissolved in citrate buffer, pH 4.5) after four weeks of dietary alternation, and both CON and HFD rats received an equivalent injection of citrate buffer only. Blood glucose concentrations, food intake, and body weights of the rats were monitored weekly. Rats with non-fasting blood glucose levels lower than 16.67 mmol/L (300 mg/dl) [18] were excluded from the DM group. Biochemical parameters (serum triglyceride, serum total cholesterol, and insulin) were measured on day 28 after injection of STZ. The homeostasis model assessment of insulin resistance (HOMA-IR) was calculated using a standard equation to examine insulin sensitivity [27]. The experiments described below were performed on day 35 after the STZ injection.

### 4.4. Pharmacokinetic Studies

We performed the pharmacokinetic studies of puerarin after oral and intravenous administration. For oral administration, the CON, HFD, and DM rats (seven rats per group) received a dose of puerarin (500 mg/kg) that was suspended in 0.5% sodium carboxymethylcellulose by gavage. For intravenous administration, all rats (*n* = 7 per group) were administered 20 mg/kg puerarin in normal saline via a tail injection. A blood volume of less than 0.3 mL was sampled via the oculi chorioideae vein before dosing at 0.17, 0.33, 0.50, 0.75, 1.0, 1.5, 2.0, 3.0, 4.0, 6.0, and 10 h after oral administration, and at 0.08, 0.17, 0.33, 0.50, 0.75, 1.0, 1.50, 2.0, 3.0, and 5.0 h after intravenous administration under light ether anesthesia. All blood samples were immediately centrifuged at 5000 × *g* for 10 min, and the plasma was collected and frozen at −40 °C until analysis.

### 4.5. Sample Preparation and Determination

One hundred microlitres of rat plasma was collected in a 1.5-mL Eppendorf tube, before 10 μL of 200 µmol/L methyl *p*-hydroxybenzoate (the internal standard, IS) was added, and the solution was mixed well. Then, 500 mL methanol was added to the precipitate protein. After vortexing for 1 min and centrifugation at 15,000 × *g* for 20 min, the supernatant was collected and evaporated to dryness under a stream of nitrogen gas at 45 °C. The residue was reconstituted in 100 μL acetonitrile by vortexing for 1 min, followed by centrifugation at 15,000 × *g* for 20 min. The supernatant (80 μL) was removed into a sample vial for HPLC analysis.

The puerarin in the samples was quantified using a Waters 2695 HPLC equipped with a 2489 UV/Vis detector (Waters, Manchester, UK) and a reversed-phase C_18_ Ecosil analytical column (250 mm × 4.6 mm, 5 μm). The mobile phase was composed of acetonitrile and ammonium acetate (2.5 mmol/L, pH 7.4) at a flow rate of 1.0 mL/min, and the column temperature was maintained at 35 °C. The eluate was monitored by absorbance at 250 nm, and the volume of sample loaded was 20 μL.

### 4.6. Preparation of Standard and Quality Control Samples

The stock solutions of puerarin and IS were prepared in methanol at a concentration of 10 mmol/L, and stored at −20 °C. The working concentration of IS was 200 μmol/L. The calibration standard samples for puerarin were prepared in blank rat plasma at concentrations of 0.1, 0.2, 0.5, 1, 2, 5, 10, 20, 50, and 100 μmol/L. The quality control (QC) samples were prepared at low (0.2 μmol/L), medium (5 μmol/L), and high (80 μmol/L) concentrations in the same way as the samples for calibration, and stored at −20 °C until use.

### 4.7. Method Validation

The selectivity of the method was determined at the lower limit of quantification (LLOQ) by comparing the blank responses of plasma from five different sources with the peak areas afforded by the LLOQ samples. The calibration curves were obtained by plotting the peak ratios of each analyte/IS versus its concentrations in plasma by using a linear least-squares regression. The intra- and inter-day precision and accuracy of the method were determined on the same day, and on five consecutive days, respectively, by using five replicates of lower quality control (LQC), medium quality control (MQC), and higher quality control (HQC) samples. The precision was calculated as percentage relative standard deviation (RSD), while the accuracy was estimated as percentage relative error (RE). The extraction recovery was assessed by comparing peak areas obtained from extracted spiked samples with those of the original spiked plasma samples. The matrix effect was evaluated by comparison between the peak areas of the post-extracted spiked QC samples and those of corresponding standard solutions. For sample stability, three levels of QC samples were determined under different conditions, including short-term stability at room temperature (25 °C) and 4 °C for 24 h, long-term stability at −40 °C for 30 days, and three freeze–thaw cycles at −40 °C.

### 4.8. RNA Isolation and Quantitative Real-Time PCR (qRT-PCR)

The qRT-PCR analysis was used to measure mRNA levels of *Ugt1a1* and *Ugt1a7* in the liver and intestines of rats. Total RNA was extracted from frozen liver and intestine samples using the TRIzol reagent according to the manufacturer’s instructions. Complementary DNA (cDNA) that was converted from 5 μg of total RNA was used. The sequences of primers are listed in Table 6. After denaturing at 95 °C for 2 min, the amplification was performed using 40 cycles of 95 °C for 15 s, and 60 °C for 1 min. Melting curves were acquired to determine the specificity of the PCR product. Glyceraldehyde 3-phosphate dehydrogenase (*GAPDH*) was used as a reference gene. The relative amount of each mRNA was normalized to its corresponding level in normal rat tissue using the 2^−ΔΔCt^ method (GenePharma, Shanghai, China).

### 4.9. Ugt1a1 and Ugt1a7 Protein Expression in Rats

Western blot analysis was performed to quantify the hepatic and intestinal protein expressions of Ugt1a1 and Ugt1a7 in diabetic and non-diabetic rats. In brief, hepatic (60 μg) and intestinal (100 μg) proteins were loaded onto a 10% sodium dodecyl sulfate (SDS)-polyacrylamide gel and subjected to electrophoresis at 100 V for 2 h. Separated proteins were transferred to a polyvinylidene fluoride (PVDF) membrane (0.45 μm), which was then blocked with 5% milk in tris-buffered saline (TBS) containing 0.01% sodium azide and 0.05% Tween^®^ 20 (TBST) for 1 h at room temperature (26 °C). Subsequently, the membranes were incubated with primary antibodies against *Ugt1a1* (1/1000), *Ugt1a7* (1/500), or *GAPDH* (1/5000) overnight at 4 °C in TBST. Afterward, the blots were washed three times with TBST, followed by incubation with horseradish peroxidase-conjugated anti-immunoglobulin G (lgG) secondary antibodies (1/100,000 dilution, Abbkine Co., Wuhan, China) for 1 h at room temperature, and then washed again. The bands were visualized using a MicroChemi 4.2 Bio-imaging System (DNRbio Co., Jerusalem, Israel), and the densitometric analyses of the immunoblots were performed using Image-Pro Plus software (Media Cybernetics Inc., Rockville, MD, USA). *GAPDH* was used as the loading control.

### 4.10. Data Analysis and Statistics

The pharmacokinetic parameters were processed by non-compartmental analysis using DAS 2.1.1 software (Mathematical Pharmacology Professional Committee of China, Shanghai, China). Data are presented as mean ± SD. Statistical differences were evaluated by one-way analysis of variance (ANOVA). A *p*-value of less than 0.05 indicated a statistically significant difference (SPSS 18.0 software, SPSS Inc., Chicago, IL, USA).

## Figures and Tables

**Table 1:** Biochemical parameters and alterations in experimental rats.

Parameters	CON	HFD	DM
Serum triglyceride (mmol/L)	2.01 ± 0.56	3.25 ± 0.34 *	4.97 ± 0.61 *
Serum total cholesterol (mmol/L)	2.54 ± 0.87	3.89 ± 0.41 *	5.12 ± 1.07 *
Initial serum glucose (mmol/L)	7.00 ± 0.47	7.34 ± 0.80	7.37 ± 0.60
Final serum glucose (mmol/L)	6.81 ± 0.93	7.71 ± 0.71	30.15 ± 4.60 **^,##^
HOMA-IR	5.29 ± 3.22	10.83 ± 1.72 *	20.60 ± 10.09 **^,##^
Initial body weight (g)	242.57 ± 24.97	258.57 ± 22.20	266.64 ± 14.90
Final body weight (g)	380.29 ± 32.89	419.5 ± 41.36 *	389.36 ± 37.52

Data were expressed as mean ± SD, *n* = 7, *p <0.05, **p <0.01 in diabetes mellitus (DM) rats versus control (CON) rats; ^#^ *p* <0.05, ^##^ *p* <0.01 in DM rats versus high-fat diet (HFD) rats. HOMA-IR—homeostasis model assessment of insulin resistance.

**Table 2:** Extraction recovery, and intra-day and inter-day precision and accuracy data from HPLC analysis of puerarin (*n* = 5).

Analyte	Concentration (μmol/L)	Extraction Recovery (%)	Intra-Day		Inter-Day		Accuracy (%)
			Mean ± SD (μmol/L)	Relative SD (%)	Mean ± SD (μmol/L)	Relative SD (%)	
Puerarin	0.2	90.81 ± 7.23	0.21 ± 0.02	8.51	0.22 ± 0.02	7.51	5.53
	50	91.62 ± 3.64	51.62 ± 2.18	4.22	51.31 ± 1.96	3.82	3.20
	80	93.61 ± 4.32	81.31 ± 4.58	5.63	81.22 ± 4.42	5.44	4.12

**Table 3:** Stability of puerarin in rat plasma samples (*n* = 3).

Storage Condition	Concentration (μmol/L)	Mean ± SD	Relative SD (%)
	0.2	0.20 ± 0.02	7.56
4 °C temperature for 24 h	50	53.21 ± 2.20	4.12
	80	82.27 ± 4.04	4.91
	0.2	0.20 ± 0.02	9.51
Room temperature (25 °C) for 24 h	50	51.94 ± 2.18	4.20
	80	80.08 ± 5.38	6.72
	0.2	0.19 ± 0.01	7.51
−40 °C temperature for 30 days	50	51.62 ± 1.88	3.65
	80	81.21 ± 3.42	4.21
	0.2	0.20 ± 0.02	8.01
Three freeze-thaw cycles	50	52.41 ± 2.31	4.41
(each at −40 °C for 24 h)	80	80.56 ± 3.78	4.81

**Table 4:** Non-compartmental pharmacokinetic parameters obtained for puerarin after its intravenous administration (20 mg/kg, approximately 48 μmol/kg) to DM, HFD and CON rats.

Pharmacokinetic Parameters	CON	HFD	DM
AUC_0–t_ (μmol·h/L)	54.84 ± 8.76	49.17 ± 8.36	35.80 ± 10.04 **^,##^
AUC_0–∞_ (μmol·h/L)	56.18 ± 8.39	50.07 ± 8.83	36.21 ± 10.17 **^,##^
MRT_0-t_ (h)	1.03 ± 0.08	0.97 ± 0.18	0.76 ± 0.08 **^,#^
t_1/2z_ (h)	0.98 ± 0.16	0.92 ± 0.17	0.75 ± 0.06
CL_z_ (L/h/kg)	0.87 ± 0.12	0.98 ± 0.18	1.41 ± 0.36 **^,##^
C_max_ (μmol/L)	75.40 ± 6.63	70.34 ± 16.04	70.35 ± 9.04
V_z_ (L/kg)	1.25 ± 0.32	1.29 ± 0.15	1.53 ± 0.37

AUC_0–t_, area under the curve of 0 to time t; AUC_0–∞_, area under the curve of 0 to time infinity; MRT_0–t_, mean residence time of 0 to time t; t_1/2z_, half time; CL_z_, clearance; C_max_, peak concentration; V_z_, apparent volume of distribution. Data were expressed as mean ± SD, *n* = 7 rats, * *p* <0.05, ** *p* <0.01 in DM versus CON rats; ^#^ *p* <0.05, ^##^ *p* <0.01 in DM versus HFD rats.

**Table 5:** Pharmacokinetic parameters of puerarin after its oral administration (500 mg/kg, approximately 120 μmol/kg) to DM, HFD, and CON rats.

Pharmacokinetic Parameters	CON	HFD	DM
AUC_0–t_ (μmol·h/L)	102.91 ± 19.14	88.96 ± 15.32	54.35 ± 4.81 **^,^^##^
AUC_0–∞_ (μmol·h/L)	106.37 ± 18.76	91.12 ± 15.70	55.12 ± 5.11 **^,##^
MRT_0–t_ (h)	2.36 ± 0.28	2.12 ± 0.25	2.07 ± 0.13
t_1/2z_ (h)	2.07 ± 0.33	1.77 ± 0.80	1.58 ± 0.10 *
T_max_ (h)	0.75 ± 0.18	0.75 ± 0.10	0.75 ± 0.15
CL_z_/F (L/h/kg)	11.57 ± 2.03	13.48 ± 2.10	21.92 ± 1.99 **^,##^
C_max_ (μmol /L)	58.64 ± 13.04	51.33 ± 5.48	24.99 ± 4.93 **^,##^
V_z_/F (L/kg)	35.10 ± 11.13	33.76 ± 14.25	49.73 ± 3.01 **^,##^

AUC_0–t_, area under the curve of 0 to time t; AUC_0–∞_, area under the curve of 0 to time infinity; MRT_0–t_, mean residence time of 0 to time t; T_max_, peak time; CL_z_/F, clearance divided by absorption fraction; C_max_, peak concentration; V_z_/F, apparent volume of distribution divided by absorption fraction. Data were expressed as mean ± SD, *n* = 7 rats * *p* <0.05, ** *p* <0.01 in DM versus CON rats; ^#^ *p* <0.05, ^##^ *p* <0.01 in DM versus HFD rats.

**Table 6:** Designed primers used for qRT-PCR analysis, and expected PCR fragment sizes.

Gene	Gene Identifier	Forward Primer	Reverse Primer	Product Length (bp)
*Ugt1a1*	NM_012683.2	5′-GCACGAAGTGGTGGTCAT-3′	5′-CGGAAGGAAAGGGTCTGT-3′	304
*Ugt1a7*	NM_130407.2	5′-AGTGTCCGTTTGGTTGTT-3′	5′-TTCCATCGCTTTCTTCTC-3′	214
*GAPDH*	NM_017008.4	5′-GCCTTCCGTGTTCCTACC-3′	5′-GCCTGCTTCACCACCTTC-3′	101

## Abbreviations

DM	diabetes mellitus
HFD	high-fat diet
CON	control
qRT-PCR	quantitative real-time polymerase chain reaction
UGT/Ugt	UDP-glucuronosyltransferase
STZ	streptozotocin
HOMA-IR	homeostasis model assessment of insulin resistance
i.v.	intravenous
p.o.	peroral
AUC_0–t_	area under the curve of 0 to time t
AUC_0–∞_	area under the curve of 0 to time infinity
MRT_0–t_	mean residence time of 0 to time t
t_1/2z_	half time
CL_z_	clearance
C_max_	peak concentration
V_z_	apparent volume of distribution
CL_z_/F	clearance divided by absorption fraction
V_z_/F	apparent volume of distribution divided by absorption fraction
